# A Rapid Systematic Review of AI‐Generated and Human‐Generated Real‐Time Feedback in Nursing Education

**DOI:** 10.1111/inr.70252

**Published:** 2026-10-26

**Authors:** Rajarajeswari Rajasekaran, Jutharat Attawet, Pratibha Bhandari, Mercy Thomas, Yunjing Qiu

**Affiliations:** ^1^ Department of Nursing School of Health Sciences Swinburne University of Technology Hawthorn VIC Australia; ^2^ School of Nursing and Midwifery Faculty of Health University of Technology Sydney Sydney NSW Australia

**Keywords:** AI, artificial intelligence, feedback, formative, human, nursing students, real time

## Abstract

**Aim:**

To explore the effects of AI‐generated and human‐generated real‐time feedback on learning outcomes and experiences among undergraduate nursing students.

**Background:**

Timely, high‐quality feedback is crucial in nursing education to support clinical reasoning, skill development, and student engagement. While human‐generated feedback remains central to pedagogy, emerging AI offers real‐time feedback that complements traditional approaches. Understanding how both interventions influence student learning is critical to informing future curriculum and clinical practice.

**Methods:**

We conducted a rapid systematic review of quantitative, qualitative, and mixed‐methods studies across major databases from 2018 to 2025. Eligible studies involved undergraduate nursing students receiving real‐time feedback generated from AI or humans. The Sample, Phenomenon of Interest, Design, Evaluation, and Research Type framework guided inclusion, and the Mixed Methods Appraisal Tool assessed methodological quality.

**Findings:**

Nine studies were included. AI‐generated tools supported timely, consistent, and individualised learning, contributing to student engagement, confidence, and skill development. Educator‐mediated feedback, including technology‐assisted and conventional human feedback approaches, supported student satisfaction, reflection, and emotional support. One study directly comparing AI‐generated and human‐generated feedback found that AI‐generated feedback offered greater consistency and objectivity, whereas human‐generated feedback provided contextual sensitivity and interpersonal connection; however, this comparison should not be generalised across the wider evidence base.

**Discussion:**

AI offers opportunities to address scalability and access challenges in feedback delivery but does not fully replicate the relational aspects of educator feedback.

**Conclusion:**

AI‐generated and human‐generated feedback demonstrate distinct yet complementary strengths. A hybrid feedback model is proposed as a plausible approach that may optimise educational outcomes in nursing; however, the included studies have not directly evaluated it, and future empirical testing is needed before it can be recommended as the most effective approach.

**Implications for Nursing:**

Combining AI‐generated and human‐generated feedback demonstrates complementary strengths, and integrating them may optimise learning outcomes.

**Implications for Health Policy:**

Investment in digital education and faculty development is needed to support scalable feedback strategies globally.

**Reporting Method:**

This systematic review adhered to relevant EQUATOR guidelines and followed the PRISMA guidelines (see Appendix 3).

## 1 Introduction

Feedback is a cornerstone of clinical nursing education and crucial for students’ professional growth in performance‐based clinical nursing education (Nilsson et al. 2025). Real‐time feedback is a fundamental component in developing clinical competence and bridging the gap between theory and practice. It strengthens clinical reasoning, technical skills, confidence, and self‐efficacy, especially in simulation‐based learning environments where immediate guidance supports mastery of complex procedures and decision‐making under pressure (Masuda et al. 2024; Yang et al. 2025).

Timely and meaningful feedback enhances students’ learning experiences and outcomes as it enables them to identify areas for improvement, reinforce safe, evidence‐based care and engage more deeply in their educational journey (Reis et al. 2024). In addition, meaningful feedback fosters the development of reflective skills, critical thinking and self‐directed learning habits, all of which are essential competencies for lifelong professional development (Archer 2010; Clynes and Raftery 2008; Yoong et al. 2023). This foundation ensures that, as students transition into registered nursing roles, they are well‐prepared to continuously evaluate and enhance their practice to deliver high‐quality patient care. International nursing organisations emphasise the importance of effective education strategies that support clinical competences, reflective practice, and patient safety (Sharplin et al. 2025). For example, the International Council of Nurses (ICN) highlights the need for innovative educational approaches to prepare a competent global nursing workforce, while the World Health Organisation (WHO) emphasises strengthening health workforce education to improve quality of care and health system resilience worldwide (Mason and Salvage 2021; WHO 2021).

Traditionally, educators, facilitators, or clinical instructors have provided feedback in nursing education through direct observation followed by verbal or written responses. While this human‐led approach remains central to nursing education, it has limitations. Factors such as variability in delivery, time constraints, and scalability issues, especially in large or resource‐constrained academic settings, challenge the sustainability of relying solely on educator‐led models (Masuda et al. 2024). These challenges are increasingly recognised across global nursing education systems, including WHO, as institutions seek scalable and innovative approaches to support the preparation of a competent nursing workforce (WHO 2025).

With the growing digital transformation of education, artificial intelligence (AI) technologies, including generative AI (Gen‐AI), are increasingly being used to supplement or provide real‐time feedback in higher education. Globally, health education institutions are increasingly exploring the use of AI‐generated feedback to enhance student engagement, personalise feedback, and support scalable educational models (Holmes and Miao 2023). These systems offer timely, personalised, and objective feedback, often delivered during or immediately after student performance. Evidence suggests that AI‐generated feedback can enhance student engagement, motivation, and academic outcomes (Venter et al. 2025). The integration of AI into nursing education is reshaping pedagogical strategies by enabling dynamic, student‐centred approaches that address both cognitive and affective dimensions of learning.

Despite the growing presence of AI in nursing education, the extent to which AI‐generated real‐time feedback has been implemented and evaluated, particularly in comparison to human‐generated feedback, remains underexplored. While human feedback is widely valued for its relational depth and contextual sensitivity, AI offers precision, immediacy, and scalability (Prakash et al. 2024). However, few studies have systematically examined the relative impacts of the two feedback sources on student learning outcomes, satisfaction, and reflective practice. This rapid systematic review addresses this gap by exploring the effects of AI‐generated and human‐generated real‐time feedback on the educational experiences and learning outcomes of undergraduate nursing students.

## 2 Aim

This review explores the impact of AI‐generated versus human‐generated real‐time feedback on undergraduate nursing students’ learning outcomes and experiences.

## 3 Methods

### 3.1 Study Design

The study used a rapid systematic review to explore current evidence on the use of AI‐generated and human‐generated real‐time feedback in undergraduate nursing education. The rapid systematic review design was selected to ensure timely synthesis of emerging technologies in research while maintaining methodological rigour (Haby et al. 2016; Tricco et al. 2017). According to the WHO's practical guide on rapid review (WHO 2021), rapid systematic reviews are streamlined approaches to synthesise evidence for informed decision‐making in health policy and systems (Hartling et al. 2015; Tricco et al. 2017). Given the emerging nature of research examining AI‐generated feedback in nursing education, predefined eligibility criteria were applied specifically to studies evaluating real‐time feedback delivered through either AI‐generated or human‐generated sources. As a result, the number of eligible studies was limited, reflecting the early stage of empirical research in this area.

### 3.2 Protocol and Registration

The review protocol was registered with the Preferred Reporting Items for Systematic Reviews and Meta‐Analysis registry (PROSPERO (york.ac.uk)). The review was conducted in accordance with a prospectively registered protocol. No substantive deviations were made from the registered eligibility criteria or synthesis approach. The research adhered to the Preferred Reporting Items for Systematic Reviews and Meta‐Analyses (PRISMA) guidelines to uphold transparency and accurate reporting (Page et al. 2021).

### 3.3 Search Strategies

A comprehensive literature search was conducted across three databases: Education Resources Information Centre (ERIC), MEDLINE and Embase, and Cumulative Index to Nursing and Allied Health Literature (CINAHL). These databases were selected in alignment with rapid systematic review guidelines, which recommend including one MEDLINE‐based database and two subject‐specific or specialised databases (Garritty et al. 2021). Search terms were hierarchically structured and combined with the Boolean operators (AND, OR) to reflect core concepts: feedback modalities, AI, undergraduate nursing students, and appropriate study designs. See the search string in Appendix 1.

Filters were applied to restrict the search to peer‐reviewed articles published in English between 2018 and 2025, reflecting recent developments in digital feedback technologies. Eligible studies included those with comparative designs, pretest–posttest designs, quasi‐experimental studies, randomised controlled trials, and qualitative studies involving focus groups or interviews. In addition to database searching, supplementary methods, such as screening the reference lists of included studies, were employed to ensure comprehensive coverage. The search strategies were tailored to each database and designed to maximise the sensitivity and relevance of retrieved results.

### 3.4 Eligibility Criteria

Two independent reviewers screened titles and abstracts against the predefined eligibility criteria. Reviewers resolved discrepancies through discussion and, when necessary, consulted a third reviewer to reach consensus. We selected studies using the Sample, Phenomenon of Interest, Design, Evaluation, Research type (SPIDER) framework (Table 1).

**TABLE 1:** SPIDER framework.

Framework	Study selection
S (Sample)	Undergraduate nursing students
PI (Phenomenon of Interest)	Real‐time feedback during simulation, skill training, or online module provided by human instructors or AI systems
D (Design)	Primary research using qualitative, quantitative, or mixed‐method designs
E (Evaluation)	Outcomes related to learning performance, engagement, confidence, satisfaction, or feedback perception
R (Research type)	Empirical research only

For this review, human‐generated feedback is defined as feedback provided by educators, lecturers, tutors, or facilitators within a learning context. AI‐generated feedback is defined as feedback automatically produced by AI systems or tools, without real‐time human input, based on student performance data, written submissions, or interaction patterns (Banihashem et al. 2024). Such feedback may be delivered through platforms utilising machine learning, natural language processing, or rule‐based algorithms. For this review, “real‐time” or “immediate” feedback was operationally defined as feedback delivered during the performance of a task or within the same learning encounter (e.g., during or immediately following a simulation, skills session or online module attempt), as distinct from delayed or summative feedback provided after a substantial time lag (e.g., at a later class, after formal grading, or days to weeks after the activity). This definition was applied equally to AI‐generated and human‐generated feedback: studies were included only where the feedback met this real‐time criterion, and no AI‐generated feedback was included based on automation alone if it was not delivered within this timeframe. We checked each included study against this definition before inclusion in Appendix 2.

#### 3.4.1 Inclusion Criteria

Studies were included if they met the following criteria: (1) involved undergraduate nursing students; (2) examined real‐time, immediate formative or AI‐generated feedback during learning activities such as simulations, online modules, unit assessments, or clinical assessment; (3) incorporated either human‐generated feedback or a direct comparison between AI‐generated and human‐generated feedback; (4) reported outcomes related to learning, clinical reasoning, decision‐making, performance, skill development, or student experience; (5) employed primary research methodologies such as quantitative, qualitative or mixed methods; (6) were published in English; and (7) were published between 2018 to 2025. The publication dates were chosen to reflect the emergence and expansion of machine learning, AI integration in education, and the increasing digitalisation of learning platforms during this period (Guan et al. 2020). Eligible studies were conducted in university‐based nursing education or clinical training settings.

#### 3.4.2 Exclusion Criteria

Studies were excluded if they: (1) focused on AI applications outside educational feedback contexts such as diagnostic tools, robotics, or administrative systems; and (2) were non‐primary publications, including editorials, opinion pieces, letters, conference abstracts, or protocols without results.

### 3.5 Selection Process

Articles retrieved from electronic databases were imported into Covidence (Veritas Health Innovation, 2025), a systematic review management platform, where duplicates were automatically removed. The first and second authors independently screened the titles and abstracts against the inclusion and exclusion criteria. To ensure consistency, the last author conducted fidelity checks on a randomly selected portion (5%) of the total records. The team discussed and resolved any discrepancies. After completing the title and abstract screening phase, the first and second authors reviewed the selected papers in full against the inclusion and exclusion criteria. To ensure fidelity, the last author checked a random 5% subset of the total records, and the team resolved any discrepancies through collaborative discussions.

### 3.6 Data Extraction and Quality Assessment

Both first and second authors reviewed a random 10% of total records to ensure data extraction consistency. The first and second authors then independently extracted data, which were subsequently cross‐checked for accuracy and consensus. Data extraction included the following information: (a) author/year; (b) country/setting; (c) study design; (d) population characteristics; (e) sample size; (f) AI‐generated feedback; (g) human‐generated feedback; (h) outcomes measured; and (i) key findings (see Appendix 2).

The authors assessed methodological quality by using the Mixed Methods Appraisal Tools (MMAT, 2018 version), a critical appraisal tool designed for evaluating qualitative, quantitative, and mixed‐methods studies (Hong et al. 2018). Two reviewers independently assessed each study against the relevant MMAT design‐specific criteria. The reviewers discussed and resolved discrepancies through consensus. Consistent with MMAT guidance, we did not assign an overall numerical quality score, as such scores may obscure important methodological strengths and limitations across appraisal domains. Instead, we recorded and reported domain‐level judgements for each of the five design‐specific MMAT criteria in Appendix 2. No studies were excluded based on methodological quality; however, identified limitations were considered when interpreting the findings and determining confidence in the synthesis.

### 3.7 Data Synthesis/Analysis

A narrative synthesis approach was employed to systematically integrate and interpret findings from studies that evaluated feedback interventions in nursing education. This method was deemed appropriate due to the heterogeneity in study designs, intervention types, and outcome measures, which precluded meta‐analysis (Campbell et al. 2020). Two reviewers independently extracted data using a structured template capturing study characteristics, intervention and comparator details, outcome measures, and principal findings. Extracted data were tabulated and organised thematically to enable systematic comparison across studies. Thematic groupings were organised by feedback delivery modality (AI‐generated vs human‐generated) to clearly identify findings.

Outcomes were categorised into key domains relevant to nursing education: clinical performance, student confidence, satisfaction, engagement and self‐regulation, and perceptions of feedback utility. To explore relationships within and across studies, the synthesis considered contextual factors such as research design, educational setting, and intervention characteristics that may have influenced observed outcomes. Where available, the synthesis extracted quantitative results such as pre–post differences and statistical significance to strengthen comparative interpretation. The team considered methodological quality and reporting limitations when assessing the robustness and credibility of findings. Throughout the synthesis process, the research team engaged in iterative discussions, peer debriefing, and critical reflection to ensure coherence, transparency, and a nuanced understanding of how different feedback sources influence nursing students’ learning outcomes.

## 4 Findings

### 4.1 Study Selection

The PRISMA flow diagram details the study selection process (see Figure 1). The systematic search identified 675 records through databases and manual searches, of which 82 were duplicates. After screening titles and abstracts (*n* = 593), 468 studies were excluded for irrelevance and inaccessible full texts. We retrieved the remaining 64 articles for full‐text review to assess eligibility for inclusion. Of these, 55 studies were excluded because of ineligible study characteristics, including non‐primary research designs and inappropriate populations, indications, outcomes, or settings. Following full‐text review, the nine included studies met the inclusion criteria and were subsequently appraised for methodological quality. Appendix 2 reports domain‐level MMAT appraisal findings. While most studies met the majority of quality criteria, several methodological limitations were identified, including small sample sizes, non‐randomised study designs, limited integration of qualitative and quantitative findings, and inadequate consideration of potential confounding variables. Consequently, confidence in findings related to student perceptions and satisfaction was lower than confidence in findings consistently observed across multiple study designs and settings. These findings were taken into account when interpreting the overall synthesis.

**FIGURE 1:**
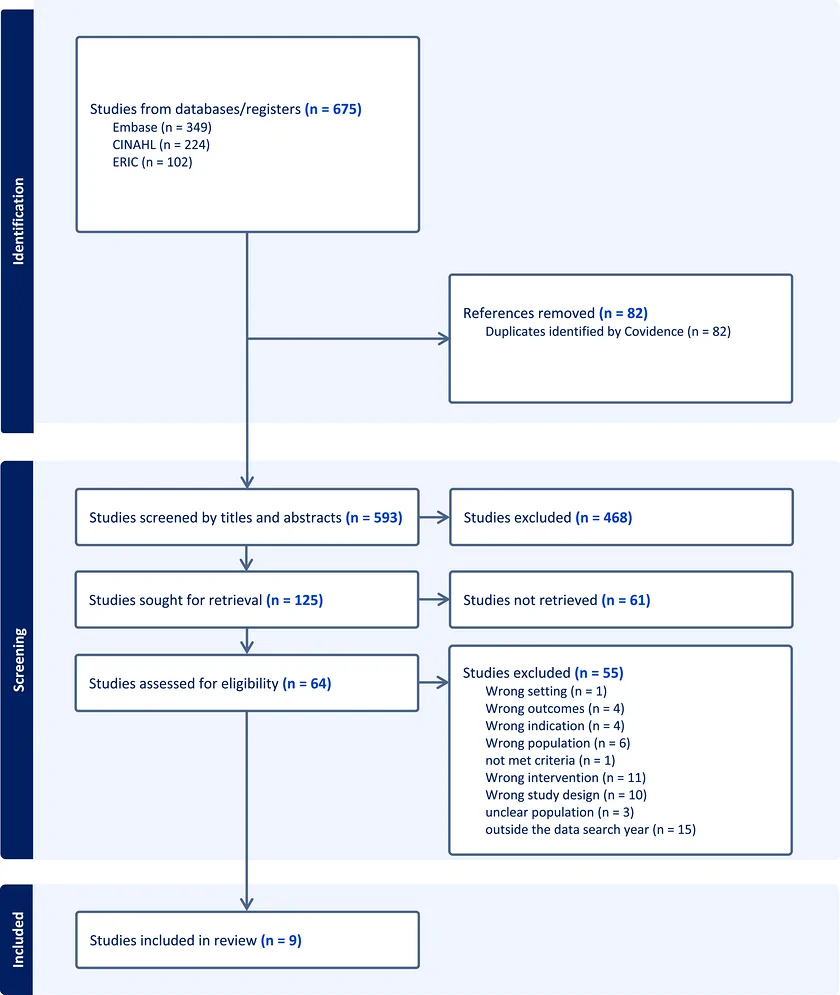
PRISMA—Preferred reporting items of systematic reviews and meta‐analysis flow diagram.

### 4.2 Study Characteristics

Two studies evaluated AI‐generated feedback: Benfatah et al. (2024) and Chen and Liou (2025), which examined ChatGPT‐driven virtual reality simulation feedback. One study evaluated automated but non‐AI‐generated feedback: Say et al. (2024), which used rule‐based scoring in online multiple‐choice tests. Four studies investigated technology‐assisted human feedback: Benfatah et al. (2025), which incorporated Video Assistant Referee (VAR) review interpreted by instructors; Lin (2022), which combined Zuvio polling with instructor verbal feedback (Zuvio is not an AI platform); Lewis et al. (2020), which used video‐based feedback with educator input; and Yang et al. (2019), which used smartphone‐supported video feedback. Two studies examined conventional human feedback: Delaney et al. (2023), which compared micro‐debriefing with standard debriefing, and Sultan and Victor (2021), which explored students’ perceptions of educator‐delivered feedback. Only findings from these nine studies are reported in Section 4.3. Citations appearing in the Discussion refer to external literature used to contextualise the review findings and are identified as such.

This rapid systematic review included nine (*n* = 9) studies from diverse geographical locations, including Australia, Taiwan, Pakistan, China, and Morocco. These studies were conducted in university, simulation centre, or clinical skills laboratory settings. Study designs included randomised controlled trials (*n* = 2), mixed‐methods designs (*n* = 1), quasi‐experimental studies (*n* = 2), cross‐sectional descriptive quantitative studies (*n* = 3), and a cross‐sectional qualitative study (*n* = 1). All studies focused on undergraduate nursing students with sample sizes ranging from 38 to 1082 participants. Two studies used AI‐generated real‐time feedback, including ChatGPT, Deepgram, OpenPose, and ChatVR‐ONCS response systems. These AI‐generated data were integrated into simulation‐based education, video‐assisted learning, and online module assessments. The remaining studies evaluated automated non‐AI feedback, technology‐assisted human feedback, and conventional human feedback. Outcomes measured across studies included clinical performance, confidence, satisfaction, engagement, self‐regulation, and, in some cases, communication skills and student perceptions of feedback quality. Most studies focused on short‐term educational outcomes immediately following training, while fewer studies evaluated sustained impacts on self‐efficacy or long‐term skill retention. Study design also differed in the strength of evidence they can support: the randomised and quasi‐experimental studies (Benfatah et al. 2024; Delaney et al. 2023) provide the strongest basis for inferring intervention effectiveness; the cross‐sectional quantitative studies (Lin 2022; Say et al. 2024; Sultan and Victor 2021) offer observational associations rather than causal evidence of effect; and the qualitative and mixed‐method studies (Chen and Liou 2025) describe participants’ experiences and perceptions of feedback, which are informative about acceptability and usability but cannot, on their own, establish effectiveness. These distinctions are maintained throughout the synthesis below.

### 4.3 Results of Synthesis

To address the diversity of feedback modalities, the included studies were classified into four categories according to the source and delivery mechanism of feedback: (1) AI‐generated feedback, (2) automated but non‐AI‐generated feedback, (3) technology‐assisted human feedback, and (4) conventional human feedback. Of the nine included studies, two investigated AI‐generated feedback interventions (Benfatah et al. 2024; Chen and Liou 2025), one examined automated but non‐AI‐generated feedback (Say et al. 2024), four evaluated technology‐assisted human feedback (Benfatah et al. 2025; Lewis et al. 2020; Lin 2022; Yang et al. 2019), and two explored conventional human feedback approaches (Delaney et al. 2023; Sultan and Victor 2021).

#### 4.3.1 AI‐Generated Feedback

Two studies evaluated AI‐generated feedback interventions (Benfatah et al. 2024; Chen and Liou 2025), and one directly compared AI‐generated and human‐generated feedback (Benfatah et al. 2024). These studies explored a range of technologies, including ChatGPT, Deepgram, OpenPose, and ChatVR‐ONCS integrated into simulation‐based training, online modules, and classroom activities. The studies reported that AI‐generated feedback integrating Deepgram speech analytics and OpenPose motion capture led to significantly greater improvements in students’ clinical performance, confidence, and satisfaction than traditional instructor‐led debriefing. Similarly, Chen and Liou (2025) reported that the ChatVR‐ONCS feedback facilitated students’ self‐correction and deeper reflection, leading to improved communication skills, increased confidence, and positive perceptions of system usability. Across both studies, students perceived the AI‐generated feedback as constructive, timely, nonjudgemental, and engaging (Benfatah et al. 2024; Chen and Liou 2025). In particular, many students appreciated its objectivity and immediacy, reporting increased motivation and reduced performance anxiety (Chen and Liou 2025). However, despite these positive attributes, the studies noted that AI‐generated feedback lacked emotional nuance and the ability to fully address the relational and interpersonal aspects of learning typically provided by human educators.

#### 4.3.2 Automated but Non‐AI‐Generated Feedback

One study examined automated but non‐AI‐generated feedback (Say et al. 2024). The intervention involved rule‐based, automated, score‐only feedback delivered after assessment activities and was compared with elaborate human feedback. Automated score‐only feedback promoted student engagement, self‐regulation, and collaboration; however, participants reported lower satisfaction than those who received detailed human feedback. These findings suggest that automated feedback can support learning behaviours efficiently but may lack the explanatory depth and personalisation associated with educator‐delivered feedback.

#### 4.3.3 Technology‐Assisted Human Feedback

Four studies explored technology‐assisted human feedback, in which technology supported feedback delivery, but educators remained responsible for interpretation, feedback provision or facilitation (Benfatah et al. 2025; Lewis et al. 2020; Lin 2022; Yang et al. 2019). Technologies included VAR‐assisted simulation review, video‐based assessment platforms, classroom response systems and smartphone‐supported video feedback. Across studies, these approaches enhanced learner engagement, reflection, and performance by providing timely and accessible feedback. Students particularly valued feedback linked to observable performance and educator guidance, indicating that technology can strengthen feedback processes without replacing the contextual and interpersonal contributions of human educators.

#### 4.3.4 Conventional Human Feedback

Two studies investigated conventional human feedback delivered directly by educators without technological mediation (Delaney et al. 2023; Sultan and Victor 2021). Delaney et al. (2023) compared micro‐debriefing during simulation with traditional post‐simulation debriefing and found no significant differences in knowledge acquisition, confidence, or satisfaction (Sultan and Victor (2021) reported that students valued clear and timely feedback but identified challenges when feedback was perceived as unclear or emotionally insensitive. More than half of participants reported feeling discouraged by negative feedback experiences, highlighting the importance of effective educator feedback practices.

Appendix 2 compares the four feedback categories: AI‐generated feedback, automated but non‐AI‐generated feedback, technology‐assisted human feedback, and conventional human feedback.

## 5 Discussion

The evidence base synthesised in this rapid systematic review comprised four feedback categories (AI‐generated, automated but non‐AI‐generated, technology‐assisted human, and conventional human feedback). However, discussion is organised around the broader distinction between AI‐generated and educator‐mediated feedback.

The synthesis of findings on AI‐generated feedback in undergraduate nursing education emphasises a notable paradigm shift in pedagogical strategies, leveraging emerging technologies to enhance student engagement, skill acquisition, satisfaction, and clinical decision‐making. Benfatah et al. (2024) found greater improvement in confidence, knowledge acquisition, and participation of nursing students who received real‐time AI‐generated feedback compared to those with traditional instructor‐led feedback. The incorporation of a variety of AI tools, from conversational agents like ChatGPT to sensor‐based systems such as OpenPose and Deepgram across simulations, online modules, and classroom environments, demonstrates AI's potential to provide immediate, personalised, and contextually relevant feedback. These tools support both the cognitive and psychomotor domains of nursing education (Benfatah et al. 2024; Rodrigues and Cruz‐Correia 2024). In addition, Lin (2022) found that a two‐tier feedback system incorporating instructor input with technology‐based tools such as Zuvio improved student concentration and satisfaction. Although Zuvio is not an AI platform, its integration in facilitating human‐generated real‐time feedback illustrates how combining technological support with educator facilitation can enhance formative assessment (Lai 2024).

These findings are also relevant within the broader global context of nursing education. Health systems worldwide are experiencing increasing workforce pressures, growing student cohorts, and limited faculty resources, creating challenges for providing timely and personalised feedback to learners (WHO 2025). International professional organisations such as the ICN emphasise the importance of strengthening nursing education technologies, including AI, which are increasingly being explored as complementary tools to support scalable and effective teaching strategies in nursing education (Sharplin et al. 2025).

Performance improvements attributed to AI‐generated feedback reflect its capacity to support active learning by enabling students to receive formative evaluation in real time. At the same time, it promotes self‐regulated learning and the refinement of clinical competencies, which leads to a significant increase in confidence (Hwang et al. 2024). However, notable limitations persist. AI tools currently lack emotional nuance and relational adaptability valued in healthcare education. Their standardised outputs may lack empathy or contextual sensitivity, which are essential elements for developing professional identity and interpersonal skills in nursing. Though AI tools offer substantial benefits for procedural and cognitive learning, they are yet to replicate the relational, ethical, and psychosocial dimensions that human educators provide.

The increasing digital transformation of higher education has accelerated the adoption of technology‐enhanced learning strategies across health professions education globally. WHO has highlighted the need to strengthen the workforce through innovation and flexible educational approaches that improve workforce preparedness and health system resilience (WHO 2025).

In contrast, the synthesis of human‐generated feedback highlighted its enduring importance for developing reflective practice and professional identity. Instructor‐led debriefing, structured formative assessment, and video‐based feedback were associated with improvements in discrete clinical skills, including vital signs measurement and aseptic technique (Delaney et al. 2023; Yang et al. 2019). Students frequently described human‐generated feedback as more adaptive and emotionally supportive, contributing to increased motivation, trust, and self‐awareness when delivered constructively. However, variability in delivery remained a recurrent challenge, as students may experience criticism or discouragement when feedback is poorly timed or unclear (Sultan and Victor 2021). These findings underscore the need to support educators in providing feedback that is both pedagogically effective and emotionally sensitive.

From a nursing practice perspective, integrating AI‐generated and human‐generated feedback offers opportunities to strengthen clinical readiness and support the transition from student to professional practitioner. The study by Zhang and Zhang (2024) offers relevant contextual evidence from the wider teacher‐education literature (external to this review's nine included studies) that suggests how AI technologies can enhance teaching support; this contextual work, rather than a review finding, helps situate how such tools might ultimately prepare nurses to deliver safe, responsive care in increasingly complex healthcare environments. Systems like OpenPose can support mastery of procedural skills such as cardiopulmonary resuscitation and safe medication‐handling techniques. Similarly, ChatGPT‐driven conversational agents can support reflective thinking, clinical reasoning, and decision‐making in scenarios that closely mirror real patient interactions (Elliott et al. 2025; Zhang et al. 2022). These functionalities respond to well‐documented constraints in nursing education, including limited faculty availability to deliver individualised feedback and the growing demand for scalable instructional methods (Wiles 2015). AI not only augments traditional teaching strategies but also expands access to consistent, timely feedback mechanisms that can sustain students’ motivation, confidence, and skill development.

Despite growing interest in AI in higher education, the limited number of studies identified in this review highlights the early stage of research on AI‐generated feedback in undergraduate nursing education. Most studies were conducted within specific institutional contexts, which may limit the transferability of findings across diverse international education systems. Future research should therefore examine the implementation and effectiveness of AI‐generated feedback across a broader range of cultural, institutional, and geographical contexts. Such research would contribute to a more comprehensive understanding of how emerging educational technologies can be integrated into nursing curricula while maintaining educational quality, ethical standards, and culturally responsive teaching practices (Almalki et al. 2025).

When combined with human‐generated feedback, these technologies can provide prompt and accessible feedback while maintaining the relational and ethical foundations of person‐centred nursing practice. This integrated approach supports both efficient learning and meaningful educational experiences in contemporary nursing education.

### 5.1 Implications for Nursing and Health Policy

The findings from this rapid systematic review have important implications for nursing educators, curriculum developers and health policymakers, navigating the evolving integration of AI in nursing education. By systematically evaluating the impact of AI‐generated and human‐generated real‐time feedback, this review offers evidence‐based insights to inform the development of scalable, student‐centred and pedagogically sound teaching strategies.

While AI offers consistency and scalability, the irreplaceable role of human‐generated feedback in fostering empathy, professional identity and context‐sensitive communication needs to be preserved. A hybrid feedback model that combines the strengths of both modalities is a plausible direction for future practice; however, because no included study directly evaluated a hybrid model, treat this as a hypothesis for future research and cautious pilot evaluation rather than a recommendation for widespread implementation. Any adoption of AI‐assisted feedback tools should be accompanied by educator oversight, staff digital‐literacy training, safeguards for data protection and student privacy, explicit attention to equity and cultural or linguistic bias in tool design and deployment, and stronger comparative research before broader rollout. To support this integration, educators must receive professional development in digital literacy and pedagogical strategies for integrating AI‐assisted tools into teaching practice.

From a policy perspective, institutional leaders and governing bodies should prioritise developing and adopting AI tools that are pedagogically robust, culturally inclusive, and ethically sound. National nursing councils, accreditation boards, and higher education institutions should work collaboratively to establish guidelines and standards for integrating AI into curriculum design, feedback delivery, and student assessment, while ensuring these technologies align with quality education and equity in nursing training.

## 6 Limitations

This review was conducted as a rapid systematic review, a design that inherently involves methodological streamlining compared with a full systematic review. Although the review followed a structured and transparent process guided by the PRISMA 2020 framework and the SPIDER tool, time constraints may have limited some elements, such as extensive manual reference checks beyond the included studies. The number of studies included was small (*n* = 9). Conclusions regarding AI‐generated feedback are based on only two studies, only one of which directly compared AI‐generated and human‐generated feedback within the same sample; the remaining evidence derives from separate, heterogeneous studies using different designs (randomised, quasi‐experimental, cross‐sectional and qualitative) and different forms of feedback (e.g., conversational‐agent, motion‐capture and video‐based systems), which limits the extent to which findings can be generalised or combined. The evidence base is also geographically limited, drawn from a small number of countries, further constraining transferability. This limited number of AI‐focused studies weakens conclusions about the effectiveness and implementation of AI‐generated feedback in undergraduate nursing education. In addition, heterogeneity in study design, intervention type, outcome measures, and feedback modalities limits the ability to conduct meta‐analysis. Lastly, definitions of ‘real‐time feedback’ and ‘AI‐generated feedback’ varied across studies, making synthesis complex. Although the included studies represented a limited range of geographical contexts, this finding reflects the emerging nature of research examining AI‐generated feedback in undergraduate nursing students. The rapid evolution of AI technologies in education has only recently prompted empirical investigation within nursing programmes.

## 7 Conclusion

This rapid systematic review shows that AI‐generated feedback can enhance clinical skills, confidence, and decision‐making in undergraduate nursing students. In contrast, human‐generated feedback remains critical for developing empathy, reflective practice, and professional identity. AI offers strengths in procedural accuracy, self‐directed learning, and scalability, whereas human input provides the relational and contextual elements essential to nursing. A blended approach integrating both may offer a promising direction for professional preparation. However, the included studies have not directly tested it, and it should be regarded as a hypothesis requiring future comparative evaluation rather than an established recommendation. Future research should therefore explore the effectiveness of AI‐generated feedback across a broader range of international educational settings to strengthen the global evidence base and support the development of scalable feedback strategies for nursing education worldwide.

## Author Contributions

Study design: RR, JA, and SQ. Data collection: RR and JA. Data analysis: RR, JA, and SQ. Manuscript writing: RR, JA, SQ, PB, and MT. Critical revisions for important intellectual content: RR, JA, SQ, PB, and MT.

## Funding Information

The authors have nothing to report.

## Conflicts of Interest

The authors declare no conflicts of interest.

## Supporting information




**Supporting File 1**: inr70252‐sup‐0001‐SuppMat.docx

**Supporting File 2**: inr70252‐sup‐0002‐SuppMat.docx

**Supporting File 3**: inr70252‐sup‐0003‐SuppMat.docx
