# Supplementary material for: A Rapid Systematic Review of AI‐Generated and Human‐Generated Real‐Time Feedback in Nursing Education
Source: Int Nurs Rev. 2026 Oct 26;73(4):e70252. doi: 10.1111/inr.70252 (PMC13697230; doi:10.1111/inr.70252)
Supplement: Supplementary file 2 [file INR-73-0-s002.docx]

**Appendix 2: Summary of the included studies characteristics: Narrative Synthesis**

|  |  |  | **Participants** | | **Intervention/Comparator** | |  |  |  |  |
| --- | --- | --- | --- | --- | --- | --- | --- | --- | --- | --- |
| **Author/Year** | **Country/**  **Setting** | **Study Design** | **Population** | **Sample size** | **Technology supported feedback** | **Human Feedback** | **Outcome Measured** | **Feedback Classification** | **Findings** | **MMAT Domain-Level Appraisal and Key Limitations** |
| Benfatah et al. (2024) | Morocco – Higher Institute of Health Sciences, Hassan First University of Settat (simulation centre) | Randomised comparative (experimental vs. control) simulation study | Undergraduate nursing students | n = 40 (20 AI group, 20 control) | AI-assisted simulation debriefing: Deepgram (speech analytics) + OpenPose (motion capture) to generate objective metrics, plus ChatGPT Q&A during debriefing  AI-generated feedback (speech analytics, motion analytics, and ChatGPT-generated response) | Traditional instructor-led debriefing without AI analytics  Facilitator-guided reflective discussion based on observation notes | • Confidence in managing asthma crises (5-point scale)  • Knowledge & clinical skill score (5-point)  • Satisfaction with debriefing (10-point)  • Participation, communication, critical reflection, clinical understanding (post-session rubric) | AI-generated feedback | AI group showed larger pre/post gains:  • Confidence +1.4 vs +0.8  •Knowledge/skills +1.3 vs +0.8  • Satisfaction +2.2 vs +1.3 (all p < 0.05) Higher participation (+0.7) & critical reflection (+0.6) than control (p < 0.05); communication & clinical-understanding differences NS | Screening questions met: Yes  MMAT Category 2 (Randomised Comparative study)    Category 2.1:Yes  Category 2.2 :Yes  Category 2.3 :Yes  Category 2.4 :No  Category 2.5 :Yes  Key limitations: small sample size (n = 40) may limit generalisability |
| Benfatah et al., 2025 | Morocco / Hassan First University of Settat (Simulation Center) | Experimental (randomized controlled simulation study) | Third-year nursing students | n = 40 (20 VAR group, 20 control) | N/A AI  Technology: Simulation with Video Assistant Referee (VAR) technology (real-time video analysis and feedback) | Simulation without VAR (standard simulation debrief) | Clinical performance: decision-making, CPR initiation, teamwork, feedback provision, communication | Technology-assisted human feedback | VAR group outperformed control (e.g., total score: 79% vs. 64%, p < 0.001). Improved rapid decision-making, CPR, and team coordination. | Screening questions met: Yes    MMAT Category 2 (Randomised control study)  Category 2.1 :Yes  Category 2.2 :Yes  Category 2.3 :Yes  Category 2.4 :Yes  Category 2.5 :Yes  Key limitation: Single-site study with a relatively small sample size |
| Chen & Liou, 2025 | Taiwan/ Nursing Clinical Skill Lab | Mixed-methods (Quantitative and Qualitative) | Nursing Students | 52 nursing students | ChatVR-ONCS  Real-time AI feedback during simulations  Automated but non-AI score only feedback (rule-based computer generated scoring) | Conventional method (implied, no control group)  Student perceptions. FGD  (human feedback not delivered, but perception was discussed) | Communication, self-confidence, newborn care skill, system usability, learning experience.  Real-time feedback from AI contributed skill development, confidence.  Participants appreciated immediate and constructive feedback received from AI during skill. | AI-generated feedback | Participants showed improved communication, confidence, skills perceived high usability.  Real-time feedback contributed to their learning experience  Suggested – AI response tone, time and expanding multilingual and cross-cultural capabilities | Screening questions met: Yes  MMAT Category 3 (Mixed Methods)  Category 3.1 :Yes  Category 3.2 :Yes  Category 3.3 :Yes  Category 3.4 :No  Category 3.5 :Yes  Key limitation: Integration of qualitative and quantitative findings was insufficiently described. |
| Delaney et al. 2023 | Australia/ Australian university | prospective, nonequivalent control group design  Control Group (Standard Simulation) and Intervention Group (Rapid Cycle Intentional Simulation- RCIS) | Second year undergraduate nursing students | 138 participants (64 in the control and 74 in the intervention group) | N/A | Control group: prebrief and debrief after the simulation. The Pendleton’s method was used during debriefing.  Intervention group: prebrief and micro-debriefing throughout the simulation. | 1. clinical deterioration knowledge  2. Satisfaction  3. self-confidence | Conventional human feedback | There were no significant differences in knowledge, confidence, or satisfaction between standard debriefing and RCIS. This suggests that end-of-simulation debriefing and micro-debriefing during simulation are equally effective. However, post-simulation debriefing limits the chance to correct mistakes, while micro-debriefing allows immediate application of feedback, enhancing learning. | Screening questions met: Yes  MMAT Category 3 (Control group study)  Category 3.1 :Yes  Category 3.2 :Yes  Category 3.3 :Yes  Category 3.4 :No  Category 3.5 :Yes  Key limitation: Non-equivalent control group design may introduce selection bias |
| Lewis et al., 2020 | Australia / Large multi-campus university | Cross-sectional survey with qualitative analysis | Third-year undergraduate nursing students | n = 731 open-ended responses (from 1277 invited) | N/A  Technology-assisted human feedback through instructor assessment of uploaded student videos | Video Assessment of Clinical Skills (VACS): student-recorded video of IV fluid hanging task with formative feedback and repeated submissions | Student satisfaction, feedback usefulness (rated 0–10), qualitative themes (flexibility, repetition, collaboration) | Technology-assisted human feedback | Students valued flexibility, self-reflection, multiple attempts, and feedback. Challenges included technology issues and inconsistent marking. Median satisfaction = 6/10. | Screening questions met: Yes  MMAT Category 1 (Qualitative study)  Category 1.1: Yes  Category 1.2 :Yes  Category 1.3 :Yes  Category 1.4 :Yes  Category 1.5 :No  Key limitation: Potential response bias and lack of reported strategies to address researcher influence |
| Lin 2022 | Taiwan/Class room, University | Quasi-experimental, one-group pretest-posttest | Nursing students | 52 nursing students | NA –AI  Technology: Two-tier instant feedback with Zuvio system and oral questions  Technology-assisted human feedback using he Zuvio response system (non AI-generated) | Yes (oral questions, peer assessment, classroom response) | Students’ concentration, satisfaction | Technology-assisted human feedback | Improved concentration, positive student perception on real-time feedback and satisfied with the feedback provision two tier method. | Screening questions met: Yes  MMAT Category 3 (Quasi-Experimental Study)  Category 3.1 :Yes  Category 3.2 :Yes  Category 3.3 :Yes  Category 3.4 :No  Category 3.5 :Yes  Key limitation: Causal inference |
| Say et al., 2024 | Australia, University | Quasi-experimental, mixed method | Nursing Students | N = 1082 (survey); subsets FGD (45)  NP1- score only feedback; NP2 – KR=EF feedback | NA /Score only feedback automated. | Detailed feedback (Knowledge result, Elaborated feedback) | Self regulation, engagement, collaboration, satisfaction | Automated, non-AI-generated feedback | Score only feedback promoted engagement, self regulation, collaboration but decreased satisfaction compared to human delivered detailed feedback. | Screening questions met: Yes  MMAT Category 5 (Quasi-Experimental Study)  Category 5.1: Yes  Category 5.2 :Yes  Category 5.3 :Yes  Category 5.4 :No  Category 5.5 :Yes  Key limitation: Limited reporting of confounding variables between comparator groups |
| Sultan 2021  Quantitative focus of human delivered only | Nursing college in Pakistan | Quantitative descriptive cross sessional | Nursing students | N = 38 | NA – AI feedback was not incorporated | Human delivered feedback – quantitative and qualitative cross sessional study on nurse educator delivered | Timing of feedback critical = 71%  Feedback threatened self-esteem – 55%  Feedback was not helpful to achieve assessment – 63%  Feedback impacted negatively feeling discouraged – 53% | Conventional human feedback | Feedback Mean SD Quantity and timing of feedback 1.81 0.49 Quality of feedback 2.09 0.39 Type of feedback 2.00 0.46 Utilization of feedback 2.14 0.51 | Screening questions met: Yes  MMAT Category: 4 (Quantitative study)  Category 4.1: Yes  Category 4.2 :Yes  Category 4.3 :yes  Category 4.4 :Yes  Category 4.5 :Yes  Key limitation: Small sample size and reliance on self-reported perceptions |
| Yang 2019 | China/ Wuxi Taihu University School of Nursing | Quantitative study/ | Second year undergraduate nursing students | 195 nursing students (65 in the video feedback group and 130 in the control group) | N/A  Technology-assisted human feedback through video-assisted performance review | Using video feedback. the intervention group (video feedback group), versus the routine teaching methods (no video feedback) | 1. 6 nursing skills assignments (bed making, aseptic procedure, vital signs measurement, oxygen therapy, catheterization, and enema)  2. General Self-Efficacy (GSE)  3.Satisfaction of feedback types | Technology-assisted human feedback | The final exam score of 4 Nursing skills (bed making, aseptic procedure, vital signs measurement, oxygen therapy) was improved in the intervention group.  The GSE scale had no significant differences.  Students were satisfied with video feedback. | Screening questions met: Yes  MMAT Category 3 (Quantitative study)  Category 3.1: Yes  Category 3.2 :Yes  Category 3.3 :Yes  Category 3.4 :No  Category 3.5 :Yes  Key limitation: Limited control of potential confounding variables between groups. |
| Note: Feedback classifications were applied according to four predefined categories: (1) AI-generated feedback, (2) automated but non-AI generated feedback, (3) technology-assisted human feedback, and (4) conventional human feedback. Classifications were determined from the full-text and feedback delivery mechanisms described by study authors. | | | | | | | | | | |
